# Supplementary material for: Please Don’t Compliment Me! Fear of Positive Evaluation and Emotion Regulation—Implications for Adolescents’ Social Anxiety
Source: J Clin Med. 2022 Oct 11;11(20):5979. doi: 10.3390/jcm11205979 (PMC9605076; doi:10.3390/jcm11205979)
Supplement: Supplementary file 1 [file jcm-11-05979-s001.zip › jcm-1952061-supplementary.pdf]

Journal of
Clinical Medicine

MDPI

Supplementary

Please Don't Compliment Me! Fear of Positive Evaluation and Emotion Regulation—Implications for Adolescents' Social Anxiety

Achilleas Tsarpalis-Fragkoulidis *, Rahel Lea van Eickels and Martina Zemp

Department of Clinical and Health Psychology, University of Vienna, 1010, Vienna, Austria
* Correspondence: achilleas.tsarpalis-fragkoulidis@univie.ac.at; Tel.: +43-676-3953360

Table S1. Direct Effects of the Structural Equation Model without Social Anxiety.

Direct Effects	*b*	BC 95% CI	β	BC 95% CI	*p*	R^2
Acceptance						.303
FPE	**-0.141**	[-0.185, -0.093]	**-0.345**	[-0.453, -0.229]	<.001	
FNE	0.060	[-0.040 0.156]	0.066	[-0.044, 0.275]	.234	
Depression	**-0.378**	[-0.508 -0.251]	-0.**318**	[-0.423, -0.209]	<.001	
Gender (F vs. M)	-0.105	[-0.304, 0.088]	-0.039	[-0.112, 0.032]	.291	
Gender (F vs. O)	-0.182	[-0.450, 0.119]	-0.046	[-0.114, 0.029]	.208	
Age	-0.033	[-0.031, 0.098]	0.039	[-0.038, 0.116]	.316	
Suppression						.335
FPE	-0.004	[-0.051, 0.043]	-0.010	[-0.140, 0.117]	.878	
FNE	0.009	[-0.083, 0.099]	0.011	[-0.102, 0.122]	.852	
Acceptance	**-0.452**	[-0.549, -0.356]	**-0.500**	[-0.597, 0.397]	<.001	
Depression	**0.144**	[0.024, 0.269]	**0.137**	[0.022, 0.247]	.021	
Gender (F vs. M)	-0.083	[-0.268, 0.110]	-0.034	[-0.111, 0.045]	.387	
Gender (F vs. O)	0.139	[-0.126, 0.388]	0.039	[-0.034, 0.108]	.288	
Age	-0.020	[-0.072, 0.032]	-0.025	[-0.094, 0.041]	.470	
Rumination						.635
FPE	0.010	[-0.024, 0.044]	0.029	[-0.068, 0.125]	.554	
FNE	**0.230**	[0.165, 0.290]	**0.298**	[0.215, 0.376]	<.001	
Acceptance	0.034	[-0.033, 0.104]	0.040	[-0.039, 0.121]	.328	
Depression	**0.608**	[0.516, 0.691]	**0.599**	[0.516, 0.670]	<.001	
Gender (F vs. M)	-0.050	[-0.186, 0.075]	-0.022	[-0.082, 0.032]	.454	
Gender (F vs. O)	0.010	[-0.202, 0.226]	0.003	[-0.057, 0.065]	.923	
Age	0.036	[-0.002, 0.076]	0.050	[-0.003, 0.107]	.079	

Notes. *b* = unstandardized coefficient, β = standardized coefficient, FPE = fear of positive evaluation, FNE = fear of negative evaluation, SUP = suppression, ACC = acceptance, RUM = rumination, DEP = depressive symptoms. Significant values are in bold.

Table S2. Indirect Effects of the Structural Equation Model without Social Anxiety.

Total and Indirect Effects	*b*	BC 95% CI	β	BC 95% CI	*p*
FPE → SUP					
Total Effect	**0.060**	[0.014;0.106]	**0.162**	[0.039;0.287]	.010
FPE → ACC → SUP	**0.064**	[0.040, 0.091]	**0.172**	[0.109;0.244]	<.001
FNE → SUP					
Total Effect	-0.018	[-0.123;0.084]	-0.022	[-0.151;0.104]	.728
FNE → ACC → SUP	-0.027	[-0.083;0.099]	-0.033	[-0.090;0.021]	.238
FPE → RUM					
Total Effect	0.005	[-0.026;0.036]	0.015	[-0.07;0.104]	.736
FPE → ACC → RUM	-0.005	[-0.016;0.004]	-0.014	[-0.045;0.012]	.340
FNE → RUM					
Total Effect	**0.232**	[0.168;0.292]	**0.301**	[0.217;0.379]	<.001
FNE → ACC → RUM	0.002	[-0.001;0.014]	-0.003	[-0.002;0.018]	.525

Notes. b = unstandardized coefficient, β = standardized coefficient, FPE = fear of positive evaluation, FNE = fear of negative evaluation, SUP = suppression, ACC = acceptance, RUM = rumination, DEP = depressive symptoms. Significant values are in bold.

Table S3. Means, Standard Deviations, and Correlations of all Study Variables and COVID-19 Burden.

Variable		*M*	*SD*	1	2	3	4	5	6	7
1.	SA	1.99	0.87	-						
2.	FNE	2.73	0.92	.664**	-					
3.	FPE	3.98	2.08	.698**	.496**	-				
4.	RUM	2.64	0.70	.561**	.571**	.449**	-			
5.	SUP	3.49	0.79	.231**	.192**	.264**	.254**	-		
6.	ACC	3.21	0.88	-.389**	-.250**	-.409**	-.298**	-.471**	-	
7.	DEP	1.53	0.71	.611**	.467**	.475**	.673**	.322**	-.404**	
8.	COV	55.85	28.69	.119**	.155**	.111**	.247**	.034	-0.91*	.202**

Notes. N = 647. **p* < .05. ***p* < .01; SA = social anxiety, FNE = fear of negative evaluation, FPE = fear of positive evaluation, RUM = rumination, SUP = suppression, ACC = acceptance, DEP = depression, COV = COVID-19 burden (VAS).

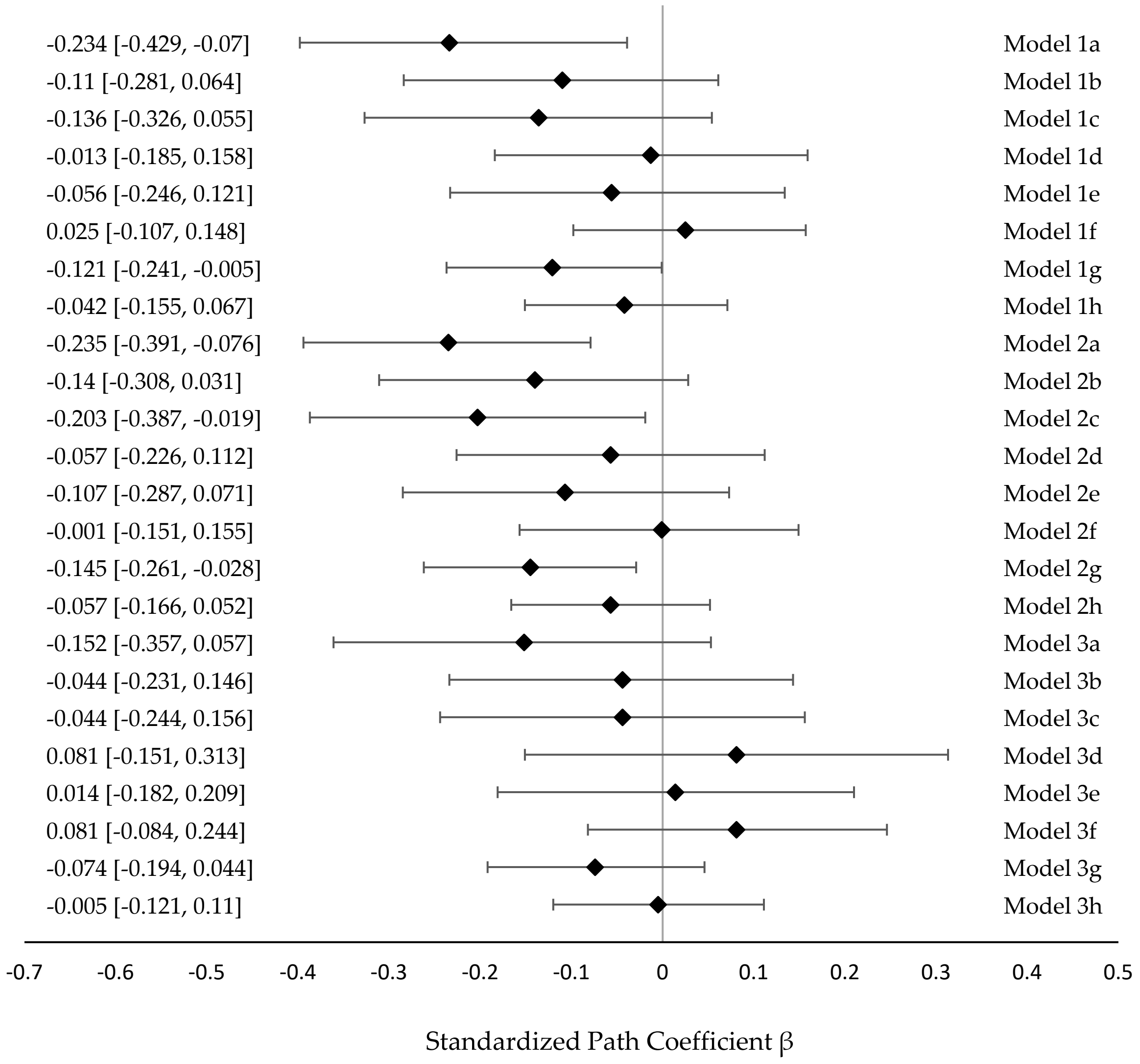

Figure S1. Coefficient Plot Displaying Standardized Coefficients of Direct Paths from all Calculated Models. Direct effect shown: Social anxiety → suppression.

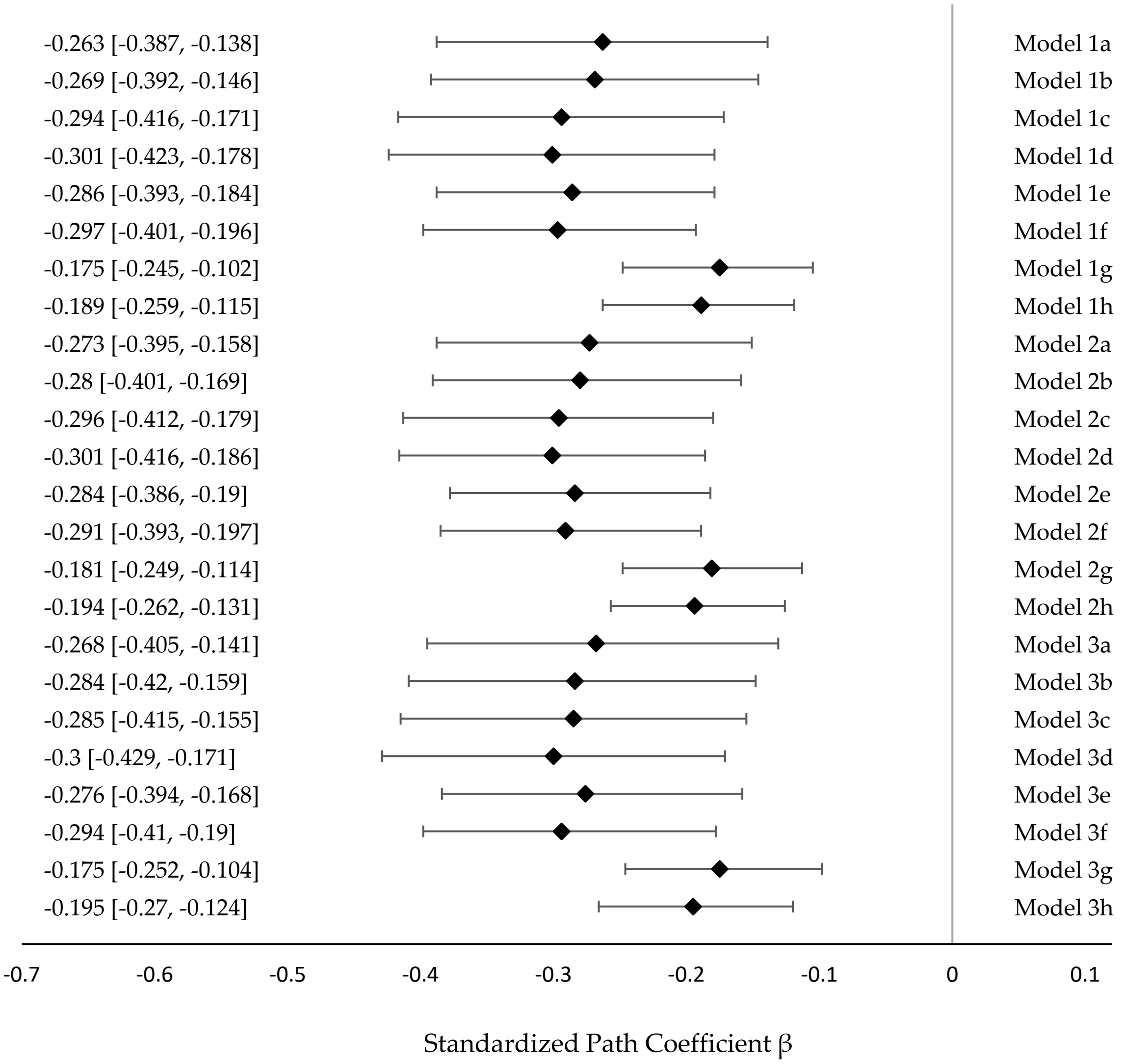

Figure S2. Coefficient Plot Displaying Standardized Coefficients of Indirect Paths from all Calculated Models. Indirect effect shown: Social anxiety → FPE → acceptance.

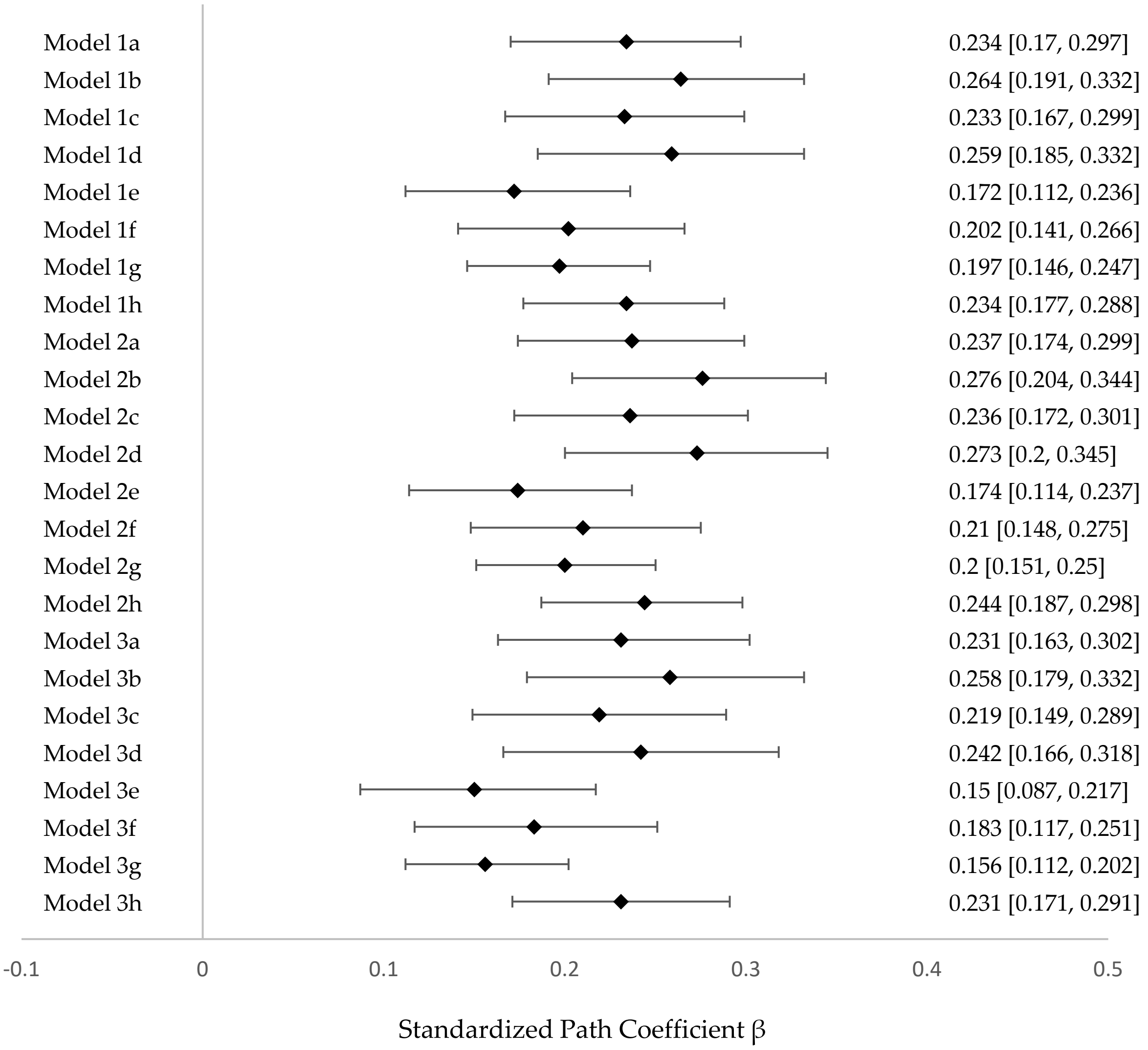

Figure S3. Coefficient Plot Displaying Standardized Coefficients of Indirect Paths from all Calculated Models. Indirect effect shown: Social anxiety → FNE → rumination.
